# Anal Adenocarcinoma Arising From a Fistula-in-Ano: A Case Report

**DOI:** 10.7759/cureus.31339

**Published:** 2022-11-10

**Authors:** Fillip N Komornik, Muhammad H Zafar, Saman Karimi, Vikas Mehta, Alejandra Perez-Tamayo, Vivek Chaudhry

**Affiliations:** 1 Colorectal Surgery, University of Illinois Chicago, Chicago, USA; 2 Pathology, University of Illinois Chicago, Chicago, USA; 3 Surgical Pathology, Mount Sinai Hospital, Chicago, USA; 4 Anatomic Pathology, University of Illinois Chicago, Chicago, USA

**Keywords:** folfox-6, extra levator abdominoperineal excision, perianal fistula in crohn’s disease, crohn’s disease (cd), fistula-in-ano, adenocarcinoma

## Abstract

Crohn’s disease (CD) is an inflammatory disease that can affect any portion of the gastrointestinal tract (GIT). Although it can present with a number of complications, perianal fistulae are among the most common consequences in patients with CD. In very rare cases, these patients can develop fistula-associated anal adenocarcinoma (FAAA). In this case report, we discuss a 72-year-old man with a long-term history of CD complicated by perianal fistulae, which failed medical and surgical management, ultimately presenting with acute anal pain in the outpatient setting. The physical examination revealed a seton traversing through a fistula surrounded by circumferential granulation tissue suspicious for malignancy. A biopsy of the tissue confirmed grade 3 mucinous-type infiltrating adenocarcinoma of the perianal skin. The patient was diagnosed with an anal verge malignancy associated with a fistula in the context of long-standing CD, and MRI staging demonstrated a T3N1 lesion with potential left inguinal node involvement. He completed neoadjuvant chemo-radiotherapy using capecitabine for five weeks with minimal tumor response, and subsequently, an abdominoperineal resection (APR) was performed with patient discharge on the fifth post-procedure day. Post-operative chemotherapy with oxaliplatin/leucovorin/fluorouracil was administered with minimal complications. Although rare, this report demonstrates the importance of consistent follow-up and mitigation of risk factors in patients with CD, along with the significance of prompt multimodal treatment in the event of developing FAAA.

## Introduction

Crohn’s disease (CD) is a relapsing, inflammatory condition of the gastrointestinal tract (GIT) characterized by transmural inflammation that can affect any portion of the GIT, with the terminal ileum being the most commonly involved [1,2]. Complications of CD include anal fissures, strictures, skin tags, abscesses, and fistulae [2,3]. Perianal fistulae are one of the more common consequences of CD and can be a complication in 20% to 54% of patients diagnosed with CD [4]. Perianal fistulae also make up 50% to 87% of all perianal complications of CD [4].

A diagnosis of CD puts patients at a greater risk of developing small bowel cancer and colorectal cancer (CRC), while the additional presence of perianal fistulae magnifies the risk of developing anal carcinoma in the long term [5]. In a meta-analysis, Jess et al. reported the standardized incidence ratio (SIR) of CRC in CD patients varied from 0.9 to 2.2, while the SIR for small bowel cancer in CD ranged from 3.4 to 66.7 [6]. The incidence of fistula-associated anal adenocarcinoma (FAAA) has been steadily increasing over the past 25 years; however, since most literature available is solely through case reports and/or case series, the true incidence and epidemiology of the disease are unknown [5,7].

In the following case report, we describe the clinical course of a gentleman presenting with longstanding perianal CD with subsequent malignant degeneration into FAAA.

## Case presentation

Patient information

The patient is a 72-year-old male with a past medical history significant for CD initially diagnosed in 1996 and subsequently was lost to follow-up without treatment. The patient did not have any active disease until 2013 when his illness was complicated by the development of recurrent perianal abscesses and a complex posterior midline fistula. Between 2013 and 2019, he was medically treated with occasional antibiotics, while multiple fistulotomies and seton placements had to be performed to manage the perianal fistulizing CD. He was started on azathioprine in October 2018 for his active disease and planned to eventually transition to adalimumab; however, the medication was ultimately never initiated due to social reasons.

A prior colonoscopy in 2018 demonstrated fragments of tubular adenoma, colonic polyps, diverticula, and erythematous mucosa in the rectum. He was an active smoker of 0.25 packs per day for about 50 years at presentation and had a history of cured hepatitis C after antiviral therapy.

The patient presented acutely with significant anal pain to the colorectal surgery outpatient clinic in early May 2020, after a recent seton placement in January 2020. He reported the anal pain to be associated with defecation but denied any fever, chills, or purulence from the non-healing fistula opening.

Clinical findings

At this outpatient clinic visit, the patient was normotensive with a slightly elevated pulse at 102 beats per minute. His abdomen was soft, non-tender, and non-distended. An anorectal examination revealed a seton in the right posterior position, multiple perianal skin tags, and butterfly-shaped granulation tissue with a firm margin surrounding the seton circumferentially in the perianal area without induration, significant pain, or purulence.

Timeline

The firm granulation tissue was biopsied to rule out malignancy; however, the results of the biopsy showed grade 3 infiltrating adenocarcinoma of the perianal skin, mucinous type. The patient was subsequently diagnosed with an anal verge malignancy associated with a fistula in the context of long-standing CD. The MRI staging demonstrated a T3N1 lesion with potential left inguinal node involvement. He completed neoadjuvant chemo-radiotherapy with capecitabine for five weeks. Post-treatment MRI showed a minimal response of the tumor to neoadjuvant therapy. In November 2020, an abdominoperineal resection (APR) was performed. He recovered well postoperatively and was discharged on postoperative day five.

Diagnostic assessment

Pathological evaluation of the biopsy specimen from the perianal skin biopsy done in the clinic in June 2020 showed invasive tumor nuclei staining positive for MLH1, PMS2, MSH2, and MSH6. Additionally, no loss of the DNA mismatch repair (MMR) protein was noted, indicating a micro-satellite stable (MSS) tumor. The tumor was also negative for mutations in the KRAS, BRAF, and NRAS genes. The tumor was staged at T3N1. The patient's carcinoembryonic antigen (CEA) level was 3.6.

Flexible sigmoidoscopy was performed shortly after to evaluate anal and rectal mucosa which revealed normal results.

**Figure 1:**
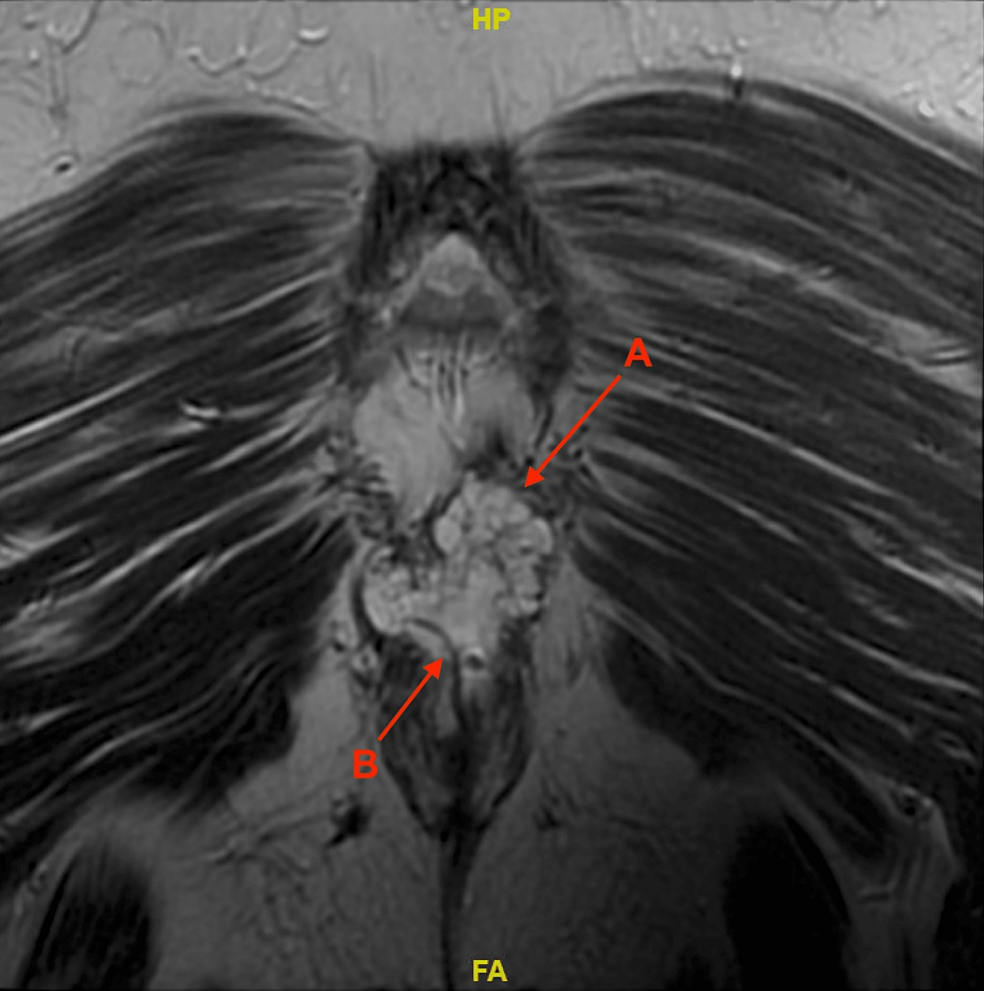
Centered within the posterior perianal region is a multilobulated septate T1 dark T2 bright mass (A) with internal enhancement measuring approximately 4.9 x 3.1 x 6.4 cm with a right anocutaneous fistula (B) traversing through the mass.

The MRI without contrast of the pelvis described the mass as located in the posterior perianal region with disruption of the internal and external anal sphincters and extension between the Coccyx and Levator Ani. The MRI also demonstrated potential left inguinal involvement, and seton transversal through the tumor was confirmed (Figure 1).

**Figure 2:**
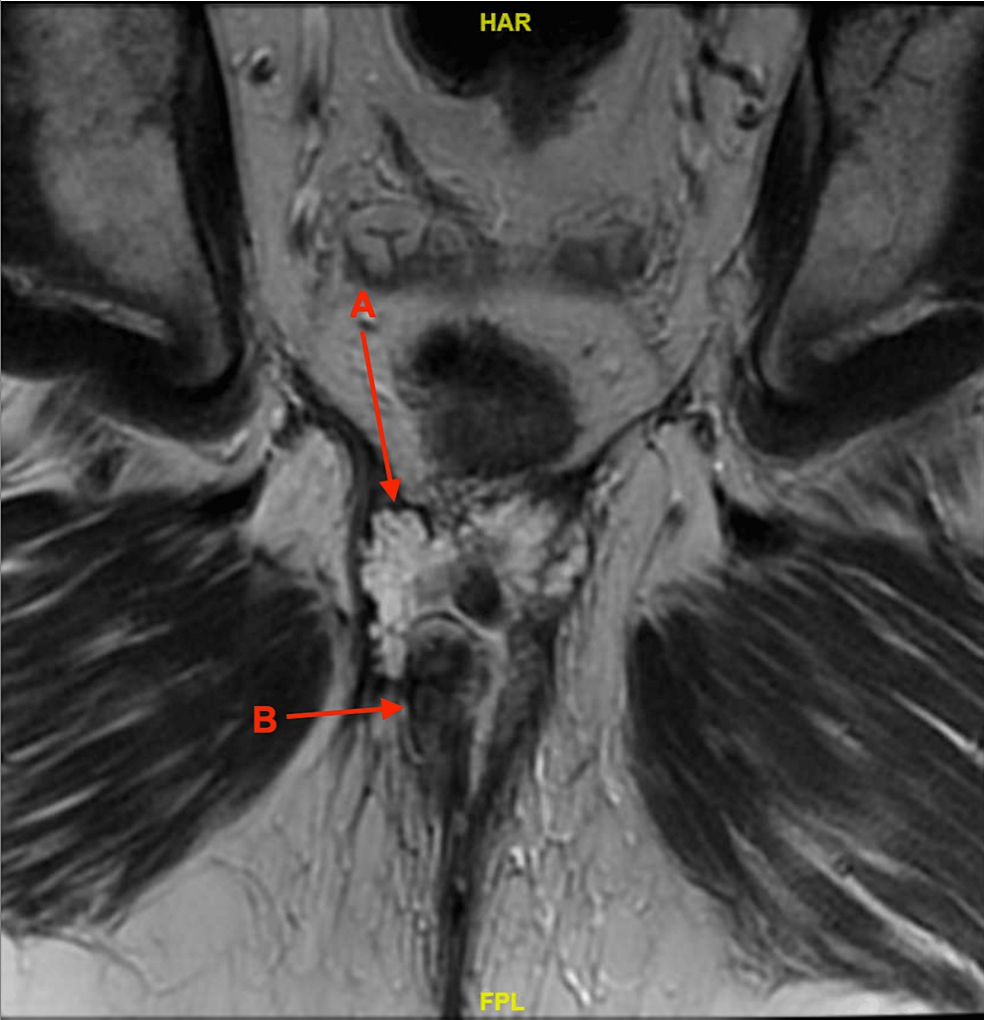
Centered within the posterior perianal region is a multilobulated septated T1 dark T2 bright mass (A) measuring approximately 3.0 x 4.2 x 5.0 cm. A right anocutaneous fistula (B) is redemonstrated traversing through the mass.

Preoperative evaluation with an MRI in October 2020 showed minimal changes relative to the initial diagnostic MRI, other than a minor decrease in left inguinal involvement (Figure 2).

An exam under anesthesia and flexible sigmoidoscopy demonstrated the adenocarcinoma as a hard anal mass fixed to the sacrum with considerable radiological changes and destruction of the anoderm.

**Figure 3:**
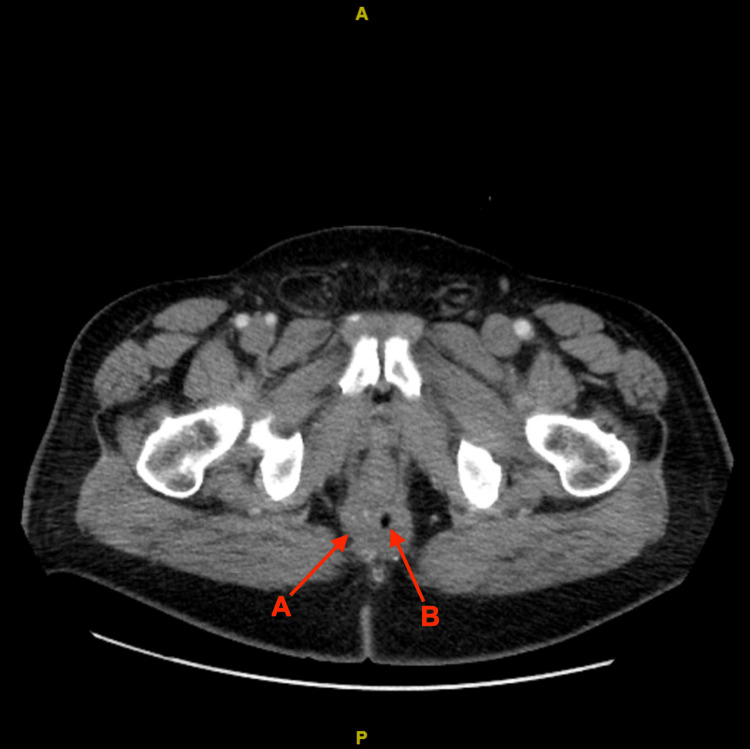
A 2.2 x 2.9 cm left perianal mass (A) is noted involving the posterior perianal fat plane, along with redemonstration of a seton suture in the right perineal region where there is a 3.9 x 3.6 cm treated abscess/fistula (B).

Post-chemoradiation CT scan of chest/abdomen/pelvis with contrast in November 2020 showed somewhat of a decrease in the size of the perianal mass, with difficulty differentiating between the mass and treated fistula/abscess. No metastatic disease was appreciated (Figure 3).

Therapeutic intervention

**Figure 4:**
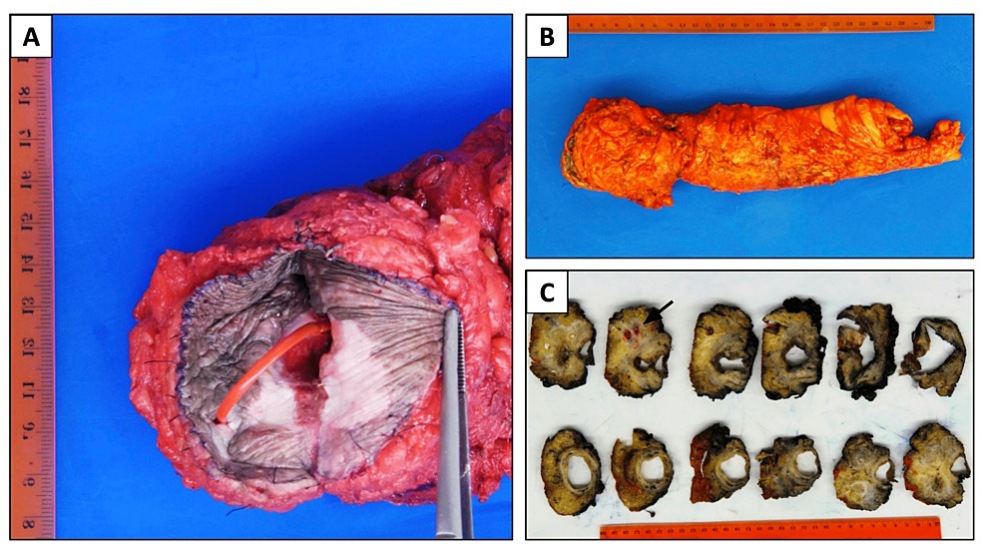
Gross examination of the specimen A: Perianal fistula tract; B: Abdominoperineal resection and partial sacrectomy, intact specimen; C: Cross-sections of the formalin-fixed specimen reveal an ill-defined, tan-white, mucoid-appearing, firm mass measuring 5.7 x 4.2 x 4.6 cm, and surrounding the fistula tract with proximal extension into the rectal parenchyma

In November 2020, the patient underwent a robotic extralevator APR with end-colostomy, en bloc distal sacral resection, and bilateral gluteal flap reconstruction of the perianal defect. Operative findings did not show evidence of peritoneal or liver metastasis. Local resection was performed with difficulty due to a large amount of fibrosis and tumor. Examination showed gross tumor invasion of the levator muscle and measured 5.7 cm in the greatest dimension (Figure 4).

**Figure 5:**
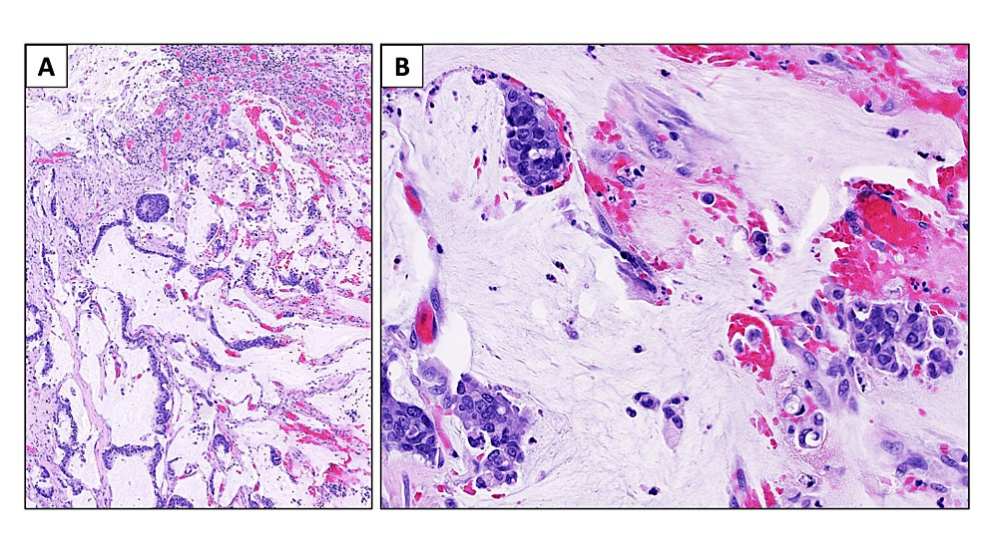
Histomorphology of the lesion A: Low magnification of the histological sections from the anal canal fistula tract reveals invasive extramucosal anal canal adenocarcinoma with mucinous features (H&E, 100x); B: High magnification of the lesion demonstrates neoplastic glands with marked pleomorphism and cytologic atypia including occasional signet ring cells, floating in a background of extravasated mucin (H&E, 400x) H&E: Hematoxylin and eosin

On histological examination, the tumor was found to be invading the muscularis propria of the rectum. Surgical margins were free of tumor invasion, and the pathology report staged the tumor as ypT3N0 (Figure 5).

Six lymph nodes were resected, and all were found to be negative for tumor invasion. Chemo-radiotherapy treatment effects were noted to be absent, and the tumor did not stain for human epidermal growth factor receptor 2 (HER2). Lymphovascular invasion was also not identified.

Following surgical intervention, the patient completed 12 cycles of chemotherapy with modified leucovorin/fluorouracil/oxaliplatin (FOLFOX-6) after evaluation by hematology-oncology. Cycle 9 was complicated by an episode of osteomyelitis that resolved with antibiotic treatment and evaluation from wound care.

Follow-up and outcomes

His immediate postoperative course was complicated by a wound seroma that was drained and packed. Unfortunately, he was re-hospitalized around one year after the surgical intervention with findings concerning for osteomyelitis. He is being surveilled for recurrence of the neoplasm with no current signs of malignancy.

## Discussion

In this case report, we have described the case of a 72-year-old man with CD who was subsequently diagnosed with FAAA and treated with a combination of chemotherapy, radiotherapy, and surgical intervention. His overall therapeutic regimen can be described as successful given the complete removal of the tumor and lack of neoplasm recurrence, and several lessons can be learned for application to future cases.

While cases of FAAA have been noted to have increased over the past few decades [5,8], reports of these types of pathologies are exceedingly rare [7,9-12]. It is thought that a combination of factors, including poor initial detection, low rates of early diagnosis, lack of effective monitoring tools, and unfavorable tumor staging at the time of surgery ultimately lead to adverse prognostic outlooks for patients diagnosed with FAAA [11,13]. Accurate diagnosis is also complicated by interference from the existing perianal fistulae in the performance of a conclusive anorectal physical examination [8].

The development of carcinoma in the context of perianal fistulae is associated with perianal disease progression for a period longer than 10 years [14]. Although a definitive mechanism for the development of this type of neoplasm is not certain, it is hypothesized that chronic inflammation combined with mucosal hyperproliferation ultimately leads to the development of the FAAA. Various literature suggests that FAAA may arise from the fistular epithelial lining or the rectal mucosa [11]. Moreover, the use of immunosuppressants and biological medications for the treatment of CD is also hypothesized to play a role in the development of these tumors; however, this idea remains disputed with conflicting evidence regarding the role of these therapies in tumor development [5,15,16]. Finally, smoking is also regarded as a risk factor for the development of FAAA [17].

When comparing the clinical course of this patient to other cases of FAAA, a number of similarities and differences can be noted. In a similar fashion to this patient, the presentation of FAAA in nearly all examined cases were associated with anal pain, a diagnosis of CD more than 20 years prior, and a corresponding long-term history of perianal fistulae. In contrast to other cases, the subject of this case report was noted to be more than a decade older than FAAA patients reported elsewhere in the available literature, at the time of presentation [5,7,10]. Regarding treatment modalities, APR was the near-universal intervention aimed at curing FAAA, with chemoradiation only being used in some cases [5,7,10]. A several-month delay in diagnosis and subsequent intervention was noted in several other cases as well [5], which could likely worsen a patient's prognosis and increase the mortality risk. In our patient, a combination of chemoradiotherapy and surgical intervention was implemented within six months of diagnosis despite the COVID pandemic. Furthermore, close monitoring of his CD via routine follow-up appointments and anorectal physical examinations in the peri-diagnostic period allowed for prompt diagnosis and initiation of treatment when his neoplasm ultimately developed.

While our patient has achieved a positive result with no evidence of recurrent disease almost two years after his APR, outcomes have varied among other cases. Results after a local resection were less optimal, with a life expectancy of only around six to 12 months when compared to a life expectancy of up to one to six years after an APR with varying rates of tumor recurrence [5,7,10]. We believe that the combination of routine monitoring of our patient's CD, early tumor detection, and prompt medical and surgical management significantly aided in the positive outcome. These factors indicate the importance of routine follow-up and prompt therapeutic intervention in patients with a concern for the development of FAAA.

## Conclusions

In summary, our experience treating this patient demonstrates the essential nature of consistent follow-up and monitoring of patients with perianal fistulizing CD concerning the development of FAAA. This case report further demonstrates the importance of prompt intervention with a combination of chemotherapy, radiotherapy, and surgical resection to optimize positive outcomes in these patients. Finally, patients should be encouraged to mitigate potentially modifiable risk factors for the development of these tumors.
